# Meharry Dental Commencement

**Published:** 1897-09

**Authors:** 


### COMMENCEMENTS.

### Meharry Dental Commencement.

The eleventh annual commencement of Meharry Dental Department of Central Tennessee College was held at the Gospel Tabernacle in Nashville, Tennessee, February 2d.

The valedictory address was given by A. L. Hill, of Kentucky. The address to the graduates was given by C. B. Wilcox, D. D., of Lafayette, Indiana.

Governor Taylor made a short and appropriate speech.

The following are the names of the dental graduates:

| | | | |
|---|---|---|---|
| J. J. Christian | West Indies | C. H. Neviells | Texas |
| A. L. Hill | Kentucky | R. B. Scott | Kentucky |
| E. B. Jefferson | Georgia | J. S. Walker | Georgia |

